# Case of Compound Ununited Fracture of the Humerus

**Published:** 1838-04-25

**Authors:** 


### CLINICAL REPORTS.

### PENNSYLVANIA HOSPITAL.

[*Reported by* HENRY H. SMITH, M. D., *resident Surgeon.*]

### *Case of Compound Ununited Fracture of the Humerus.*

JOHN D———, aged 30 years, a farmer in the interior of Pennsylvania, was run over by a loaded wagon on the 29th of May, 1837; the wheel passed across the arm, fracturing the humerus very obliquely, and bruising the soft parts to a considerable extent; a tight bandage was applied several hours after by a quack, and kept on for three days, when he was induced to go some distance to consult a physician, who cut the bandages off and applied poultices to the arm for a week. At the end of this time, a portion of the integuments around the fracture were removed, owing to mortification. This made a wound of an inch and a half in diameter, through which the ends of the bone projected; the arm was placed in a sling without splints, and ordered to be kept quiet. In this state the man remained until the middle of July, when he determined to visit the city, and was admitted into the hospital on the 10th of August, near ten weeks after the accident. A wound, the size of a dollar, existed at the point of fracture, through which the lower fragments, free of periosteum, for one inch projected; no union of the bone had taken place, although much callus was thrown out around it. The elbow was partially anchylosed, and he was unable to move the arm at all; a poultice was at once applied to the wound, a rectangular splint placed on the inside, two straight splints from the elbow to the shoulder, on the back and outside of the humerus.

*August* 13th. Arm easy, slight discharge from the wound. Upon seizing the projecting piece of bone with the forceps, it was easy to move the lower end of the humerus near the joint.

*August* 16th. Upon a consultation it was determined to await the exfoliation of the projecting portion of the bone, continue the treatment, varying the angle of the splint, and making motion at the elbow every other day. This treatment was persevered in until September 10th, the bone and wound being occasionally touched with lunar caustic, and repeated efforts being made to remove the bone with the forceps.

*September* 15th. Bone becoming loose; hardness around the wound disappearing.

*September* 29th. Nearly four months after the injury, an inch and a half of the shaft of the humerus was removed by the forceps; some union at the fracture; partial anchylosis of the elbow. The wound was dressed with cerate, and an angular splint applied to the *front* of the arm, with three straight splints from the elbow up.

*October* 16th. Wound healing, fracture tolerably firm, motion at the elbow much increased.

*October* 30th. Another small piece of the bone was removed to-day; the wound is still somewhat open, motion improving.

*November* 22d. The same treatment has been pursued since last date; another small piece of bone removed.

*November* 25th. Wound healed; arm straight and firm, with nearly perfect motion at the elbow; the wrist and fingers entirely free from stiffness. The patient is, however, unable to bring the fore arm near to the arm, on account of the adhesion of the biceps, &c. to the humerus.

*November* 27th, 1837. Seventy-seven days after admission, the man was discharged, being able to carry a considerable weight in his hand.

